# The association of eight-year trajectories in total, cognitive-affective, and somatic depressive symptoms with incident stroke: a 10-year follow-up study using HRS and ELSA cohorts

**DOI:** 10.3389/fnagi.2025.1733007

**Published:** 2026-01-09

**Authors:** Haining Zhang, Jiange Chen, Yuntian Ye, Hongyi Wang, Shun Fan, Wei Zhang, An Bao, Huanan Li, Jingui Wang

**Affiliations:** 1 Department of Tuina, First Teaching Hospital of Tianjin University of Traditional Chinese Medicine, National Clinical Research Center for Chinese Medicine, Tianjin, China; 2 Department of Orthopedics, First Teaching Hospital of Tianjin University of Traditional Chinese Medicine, National Clinical Research Center for Chinese Medicine, Tianjin, China

**Keywords:** cognitive-affective depressive symptoms, cohort studies, dynamic trajectories, follow-up studies, mid-to-late life depression, risk factors, somatic depressive symptoms, stroke

## Abstract

**Background:**

Earlier research has documented an association between depressive symptomatology and heightened stroke risk. However, prior work largely assessed depressive manifestations at isolated time points and failed to differentiate symptom subtypes. This investigation seeks to characterize the longitudinal progression of depressive symptoms via repeated measurement and explore their link to stroke risk by considering total depressive symptoms alongside cognitive-affective and somatic dimensions.

**Methods:**

This prospective cohort study included individuals aged ≥ 45 years from the Health and Retirement Study (HRS) in the United States and the English Longitudinal Study of Ageing (ELSA) in the United Kingdom, excluding those with a history of stroke during the exposure period. Depressive symptoms were measured using the 8-item Center for Epidemiologic Studies Depression Scale (CES-D) across four biennial assessments. Individuals were categorized into five distinct depressive symptom trajectories: consistently low, decreasing, fluctuating, increasing, and consistently high, based on assessment scores. Over a subsequent decade of follow-up, incident strokes were identified through self-reported physician diagnoses. The analyses incorporated adjustments for demographic factors (sex, age, etc.), health-related behaviors (smoking, drinking, etc.), and health status covariates (hypertension, diabetes, etc.). Cox proportional hazards regression models generated hazard ratios (HRs) and 95% confidence intervals (CIs) to evaluate links between trajectories of total depressive symptoms, cognitive-affective and somatic subtypes, and stroke occurrence.

**Results:**

The final cohort included 10,011 participants (63.3% female; mean age 60.2 years). During the 10-year follow-up, 720 incident strokes were recorded. Analyses demonstrated that, after adjusting for the aforementioned demographic and health-related confounders, relative to the consistently low trajectory, participants with fluctuating (HR = 1.24, 95% CI: 1.01–1.52), increasing (HR = 1.31, 95% CI: 1.03–1.67), and consistently high (HR = 1.42, 95% CI: 1.03–1.97) total depressive symptom trajectories exhibited significantly elevated stroke risk. Conversely, the decreasing trajectory (HR = 1.11, 95% CI: 0.85–1.45) did not significantly impact stroke risk. Furthermore, an increasing trajectory of cognitive-affective depressive symptoms (HR = 1.43, 95% CI: 1.13–1.82), alongside fluctuating (HR = 1.27, 95% CI: 1.03–1.55) and consistently high (HR = 1.97, 95% CI: 1.42–2.74) somatic depressive symptom trajectories, were each significantly associated with heightened stroke risk. Critically, the consistently high somatic trajectory demonstrated the most robust association with stroke.

**Conclusion:**

Trajectories of total depressive symptoms marked by escalation, instability, or sustained elevation exhibited significantly elevated stroke risk. In contrast, individuals displaying decreasing depressive symptoms exhibit stroke risk comparable to those maintaining consistently low levels. Specifically, an ascending trajectory of cognitive-affective symptoms, alongside unstable and persistently elevated trajectories of somatic symptoms, are linked to increased stroke risk. These findings emphasize the clinical importance of monitoring dynamic changes in depressive symptoms and their subtypes for stroke prevention. Future investigations should elucidate underlying mechanisms to refine identification and intervention strategies for high-risk populations.

## Introduction

As the second leading contributor to global mortality and the third primary cause of disability-adjusted life years (DALYs) (GBD 2017 Dalys and Hale Collaborators, 2018), stroke also ranks as the fifth leading cause of death within the United States (Virani et al., 2020). Epidemiological evidence indicates that the global lifetime probability of experiencing a stroke from age 25 has progressively risen over the past thirty years, currently approximating 24.9% (Meza et al., 2020). Amidst accelerating population aging and extended lifespans, the cumulative economic burden and societal consequences of stroke are anticipated to intensify (Marsh and Keyrouz, 2010). Consequently, pinpointing stroke risk determinants is essential for devising effective preventive and mitigation approaches. Established classical risk factors like smoking and diabetes are recognized (Liu et al., 2021); however, these factors alone do not comprehensively explain all stroke risk. Recent years have seen amplified focus on stroke risk factor inquiry, accumulating robust evidence suggesting that depressive symptoms can augment stroke vulnerability (Glymour et al., 2012; Meza et al., 2020). Meta-analyses consistently report associations between depression/depressive symptomatology and a 31% to 45% elevation in prospective stroke risk (Barlinn et al., 2014; Li et al., 2015). A meta-analysis including 17 prospective studies showed a significant positive association between depressive symptoms and subsequent stroke risk (pooled relative risk = 1.34, 95% CI: 1.17–1.54) after adjusting for potential confounders such as smoking, hypertension, and diabetes. This suggests that depressive symptoms are an independent risk factor for increased stroke risk, independent of confounders such as smoking and comorbid conditions (Dong et al., 2012). Moreover, research by Gilsanz et al. (2017) revealed that older adults enduring depression for a 2-year duration faced a 65% higher stroke risk relative to their non-depressed counterparts.

Contemporary research furnishes substantial evidence that depressive symptoms exhibit dynamic patterns across the lifespan, characterized by periods of remission and recurrence. Nevertheless, the majority of earlier investigations concentrated predominantly on the mere presence or short-term persistence (≤ 2 years) of depressive symptoms concerning incident stroke, frequently neglecting long-term dynamic fluctuations, specifically depressive symptom trajectories (Gilsanz et al., 2015, 2017; Glymour et al., 2012; Liu et al., 2021; Meza et al., 2020). Certain individuals experience persistent, chronic depression, while others may encounter transient symptom exacerbations precipitated by stressors. Additionally, some may suffer symptom relapse following a period of remission. Failure to longitudinally track depressive symptom progression may yield an incomplete comprehension of their association with incident stroke. Furthermore, depressive symptoms encompass multifaceted dimensions, including cognitive-affective and somatic subtypes (Iob et al., 2020). A scarcity of research probes the connection between distinct facets of depressive symptoms and cardiovascular/cerebrovascular diseases. Existing inquiries predominantly rely on static analyses conducted at discrete time points. Ongoing debate surrounds the relationship between cognitive-affective versus somatic depressive symptoms and cardiovascular/cerebrovascular disease risk (de Miranda Azevedo et al., 2014; Hoen et al., 2010a,b; Martens et al., 2010; Meijer et al., 2011; Norton et al., 2021; Wium-Andersen et al., 2019). Research specifically examining the correlation between particular depressive symptom dimensions and stroke incidence remains insufficient. Consequently, the impact of cognitive-affective and somatic depressive symptoms on stroke occurrence is not fully elucidated. Investigating potential associations between longitudinal depressive symptom trajectories and stroke events may yield more precise causal inference.

This study aims to address the existing research gap by examining the dynamic trajectories of depressive symptoms over time, including distinct subtypes, and exploring their association with stroke risk. Using data from the Health and Retirement Study (HRS) and the English Longitudinal Study of Ageing (ELSA), we conducted an 8-year foundational assessment of the trajectories of total, cognitive-affective, and somatic depressive symptoms, and their relationship with stroke risk over the following ten years. The analyses accounted for adjustments in key demographic factors (such as sex and age), health-related behaviors (including smoking and drinking), and health status covariates (such as hypertension and diabetes), providing a comprehensive evaluation of these associations.

## Materials and methods

### Study population

The investigation utilized data from HRS and ELSA, representative longitudinal datasets of aging populations in the United States and United Kingdom, respectively. Both employ standardized questionnaires and analogous measurement instruments to conduct biennial participant interviews, evaluating domains such as physical health, mental health, economic status, and other pertinent variables. This methodology facilitates examination of the relationships between depressive symptoms, their long-term trajectories, and the incidence of stroke.

**FIGURE 1:**
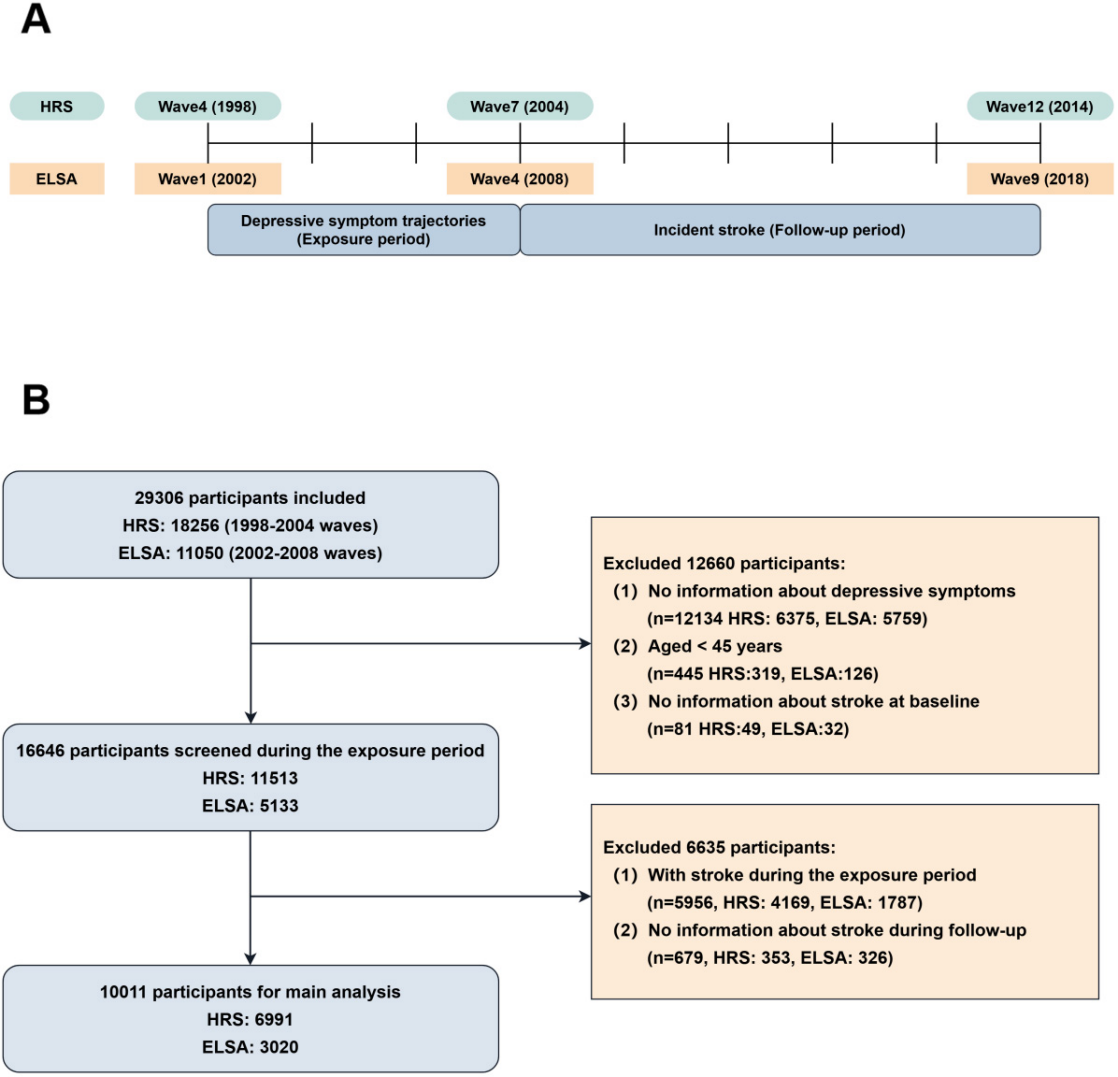
Timeline and sample selection flow of the present study. **(A)** Current study timeline. The exposure period was defined as waves 4 to 7 of HRS and waves 1 to 4 of ELSA, whereas the follow-up period included waves 8 to 12 of HRS and waves 5 to 9 of ELSA. **(B)** Process for selecting samples in HRS and ELSA according to inclusion and exclusion criteria.

We integrated data spanning HRS waves 4 to 12 (1998–2014) and ELSA waves 1 to 9 (2002–2018) to investigate the association between depressive symptom trajectories and incident stroke. HRS wave 4 (1998) and ELSA wave 1 (2002) served as baseline assessments, with subsequent waves monitored up to wave 7 for HRS and wave 4 for ELSA. Depressive symptom trajectories were evaluated across these baseline and subsequent waves, constituting the exposure period. HRS waves 8 to 12 and ELSA waves 5 to 9 were designated as the follow-up period for ascertaining incident strokes. A study timeline is depicted in Figure 1A.

### Exclusion criteria

Exclusion criteria were: (1) age below 45 years; (2) prior stroke history or stroke during exposure period; (3) incomplete depressive symptom trajectory data throughout the exposure period; and (4) missing data on stroke events during follow-up. Per World Health Organization classification, individuals aged ≥ 45 are considered middle-aged and older adults. Excluding younger participants concentrated the study on individuals more susceptible to age-related physiological and social changes potentially contributing to depressive symptoms and incident stroke. Excluding participants with prior stroke history or stroke during exposure ensured the study specifically assessed the relationship between depressive symptom trajectories and new incident stroke occurrence. Furthermore, excluding participants with missing depressive symptom or incident stroke data during follow-up aimed to minimize the influence of data imputation on overall results. A flowchart detailing the sample selection process is presented in Figure 1B.

### Primary exposures

Depressive symptoms were assessed using the validated, modified 8-item Center for Epidemiologic Studies Depression (CES-D) scale (Schlechter et al., 2023), recognized as reliable for measuring depressive symptoms in older adults (Karim et al., 2015). During biennial interviews, participants reported their experience of eight symptoms over the preceding week, with responses recorded dichotomously (yes/no) (Schlechter et al., 2023; Steffick et al., 2008). Symptoms included: (1) feeling depressed; (2) perceiving everything as an effort; (3) restless sleep; (4) feeling happy; (5) feeling lonely; (6) enjoying life; (7) feeling sad; (8) inability to get going. The total score summed affirmative responses, with reverse scoring applied to the two positively worded items (feeling happy, enjoying life), yielding a range of 0–8. Consistent with prior research, a score ≥ 3 defined clinically significant depressive symptoms in older adults (Lee et al., 2021; Soh et al., 2022). Depressive symptoms were classified into two subtypes: cognitive-affective and somatic. The cognitive-affective subtype encompassed items such as “Felt depressed,” “Felt lonely,” “Felt sad,” plus reverse-scored responses to “I was happy” and “I enjoyed life.” Conversely, the somatic subtype included items such as “Everything was an effort,” “Sleep was restless,” and “Could not get going,” acknowledged as physical manifestations of depression, particularly among older adults (Fan et al., 2025; White et al., 2017). A bifactor model of depressive symptoms, validated by confirmatory factor analysis (CFA) in prior research, effectively distinguishes these two domains, demonstrating adequate discriminant validity (Frank et al., 2020).

Utilizing CES-D scores from HRS waves 4–7 and ELSA waves 1–4, five depressive symptom trajectories were identified: consistently low, decreasing, fluctuating, increasing, and consistently high (Jia et al., 2025; Soh et al., 2022). These trajectories were defined based on symptom scores recorded at four consecutive time points. The “consistently low” trajectory comprised individuals whose scores consistently remained below the clinical threshold (< 3) at all assessments, representing no depressive symptoms or persistently mild symptoms. Conversely, the “consistently high” trajectory comprised individuals whose scores consistently met or exceeded the threshold (≥ 3) across all assessments, indicating sustained significant symptoms. The “decreasing” trajectory included individuals with initially high scores (≥ 3) showing an overall decline, culminating in scores below the initial value and not exceeding the second assessment score, thereby excluding individuals with rebound during assessments two and three. In contrast, the “increasing” trajectory pertained to individuals with initial scores below threshold (< 3) exhibiting an upward trend, culminating in final scores exceeding the initial value and not lower than the second assessment score, excluding individuals showing a decrease during assessments two and three. The “fluctuating” trajectory encompassed individuals demonstrating irregular variations in symptom severity across the four time points, lacking a consistent unidirectional pattern. The consistently low trajectory served as the reference group. Detailed trajectory definitions are in Supplementary Table 1. CES-D scores for cognitive-affective and somatic domains were dichotomized using the 75th percentile as a threshold (CES-D ≥ 2 indicating significant symptoms within each domain) (Chu et al., 2023; Lu et al., 2024). Trajectories for somatic and cognitive-affective symptoms were classified identically, with a threshold of ≥ 2 indicating significant symptoms.

### Stroke outcomes

Incident stroke was ascertained using data from HRS waves 8–12 and ELSA waves 5–9. Events were identified via self-reported or proxy-reported physician diagnoses, based on the question: “Has a doctor ever told you that you had a stroke?” An affirmative response by the participant or proxy classified a stroke event. For participants reporting stroke in a prior wave, confirmation was sought in subsequent waves. Retrospective corrections were applied if participants disputed prior self-reports. Neither dataset systematically recorded transient ischemic attacks or categorized stroke types, nor provided information on stroke subtypes. However, HRS self-reported strokes exhibit strong concordance with Medicare/Medicaid records coded using the International Classification of Diseases (sensitivity 74%, specificity 93%). Prior HRS-based research demonstrated that relationships between established risk factors and self-reported stroke incidence align closely with findings from clinically verified stroke studies (Glymour and Avendano, 2009).

### Covariates

Covariates were carefully selected to account for potential confounding between depressive symptoms and stroke risk. They encompassed baseline self-reported: (1) Demographic/socioeconomic: Age (continuous), gender (male/female), race (White/non-White), highest education (high school and below/some college/college and above), marital status (married/partnered, separated/divorced/widowed, never married). (2) Health behaviors: Body mass index (BMI, continuous), alcohol consumption (never/ever drinkers), smoking status (never/ever smokers), vigorous physical activity frequency (more than once per week/once per week or 1–3 times per month/never). (3) Health conditions: Physician-diagnosed heart disease, hypertension, diabetes, cancer (yes/no).

**FIGURE 2:**
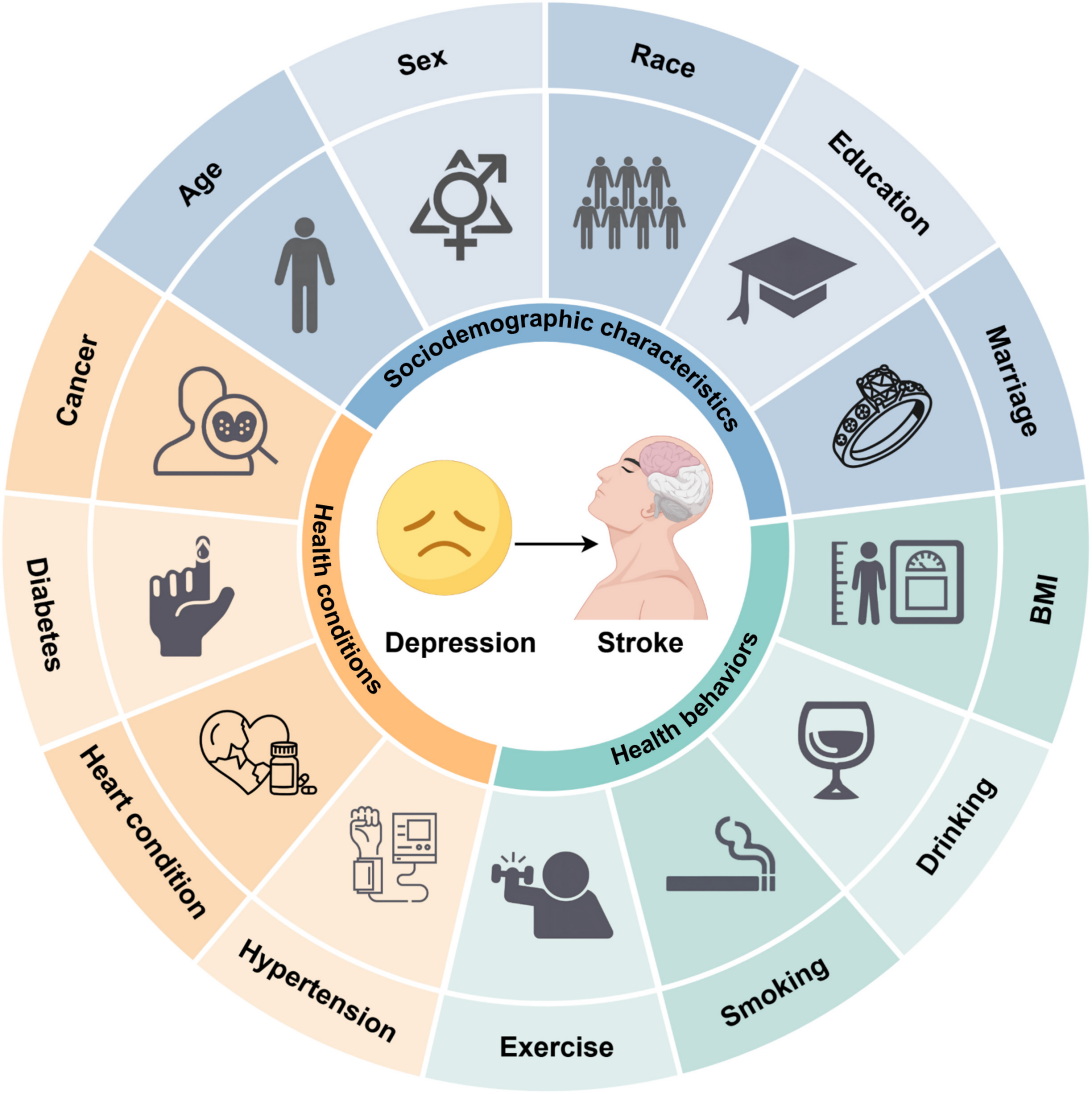
Diagram illustrating covariate classification employed in this study. The main covariates include three categories: sociodemographic characteristics, health behaviors, and health conditions.

These covariates are crucial for controlling confounding when evaluating the association between depressive symptom trajectories and incident stroke. Self-reported health conditions in HRS exhibit substantial concordance with medical records, and prior HRS research established strong external validity for these health behavior measures (Fisher et al., 2005; Glymour and Avendano, 2009; Jenkins et al., 2008; Okura et al., 2004; Wallace and Herzog, 1995). Covariate classification is illustrated in Figure 2.

### Statistical analysis

Baseline demographic data were analyzed descriptively, stratified by depressive symptom trajectories. Continuous variables presented as means (standard deviations, SD) or medians (interquartile ranges, IQR); categorical variables as frequencies (percentages). “Time” was defined as the interval from follow-up initiation to the first incident stroke or follow-up conclusion, whichever occurred first. Follow-up was censored at the participant’s last completed survey. Schoenfeld residual tests evaluated covariate effect temporal constancy; absence of association between residuals and time satisfied the proportional hazards assumption. Consequently, Cox proportional hazards models estimated hazard ratios (HRs) and 95% confidence intervals (CIs) to examine associations between depressive symptom trajectories and stroke risk. The consistently low trajectory served as the reference category in all four Cox models: (1) Model 1: Adjusted for trajectory and age. (2) Model 2: Additionally adjusted for sociodemographics (gender, race, education, marital status). (3) Model 3: Additionally adjusted for health behaviors (BMI, alcohol, smoking, physical activity). (4) Model 4: Additionally adjusted for health conditions (hypertension, heart disease, diabetes, cancer). Missing covariate data were handled using multiple imputation by chained equations (MICE) in R. Maximum iterations per dataset were 50, with five imputation rounds generating five datasets; the first complete dataset was analyzed.

Six sensitivity analyses evaluated robustness: (1) Discrete-time models: Reanalyzed Models 3 & 4 using discrete-time survival models to assess HRs across follow-up intervals (Huang et al., 2024). (2) Logistic regression: Reanalyzed Models 3 & 4 using logistic regression to compare single-time-point depressive symptoms vs. incident stroke association. (3) Stricter “consistently high” cutoff: Applied a stricter threshold for defining the consistently high trajectory and reanalyzed Models 3 & 4. (4) Refined trajectory definitions: Implemented stricter criteria for increasing/decreasing trajectories by excluding participants reporting exclusively high/low symptoms at the final exposure wave. (5) Added covariate (income): Incorporated income as a covariate and reanalyzed all four trajectory models. (6) Extended follow-up: Prolonged follow-up endpoints to HRS waves 13, 14, 15 and reanalyzed Models 3 & 4.

Statistical analyses used R software (v4.5.1). Two-tailed hypothesis tests defined statistical significance at *P* < 0.05.

## Results

Following exclusion criteria, 10,011 participants were included in the final analysis, with data spanning a 10-year follow-up. Within this cohort, 720 incident strokes were identified. Analyses focused on correlations between depressive symptom trajectories and incident stroke incidence, demonstrating significant impacts of various symptom subtypes and trajectories on stroke risk.

Tables 1, 2 present baseline demographic characteristics, health behaviors, and health conditions of the merged sample, stratified by total depressive symptom trajectory. At baseline, mean age was 60.2 years; 63.3% female; 88.8% White; 56.2% had high school education or less. Health behavior analysis: mean BMI 27.7 kg/m (Virani et al., 2020); 55.3% ever smokers; 66.7% ever drinkers; 53.9% reported no vigorous physical activity. During exposure, 62.5% exhibited a consistently low trajectory, with a notably higher proportion married/partnered (79.7%). Conversely, a substantial proportion (77.4%) within the consistently high trajectory had high school education or less. Regarding health conditions, prevalence of underlying issues was markedly higher among participants in the consistently high trajectory: 12.5% heart disease, 42.1% hypertension, 10.1% diabetes, 6.5% cancer.

**TABLE 1:** Initial characteristics of study participants at the beginning of the exposure period.

Variables	Overall sample	Depressive symptoms trajectory group				
		Consistently low	Decreasing	Fluctuating	Increasing	Consistently high
Individuals, *n* (%)	10,011	6,252 (62.5)	903 (9.0)	1,582 (15.8)	858 (8.6)	416 (4.1)
**Sociodemographic characteristics**						
Age, y, mean (SD)	60.2 (7.5)	60.4 (7.4)	59.6 (7.5)	60.0 (7.4)	60.6 (7.9)	59.9 (7.9)
**Sex, *n* (%)**						
Female	6,333 (63.3)	3,601 (57.6)	644 (71.3)	1,123 (71.0)	631 (73.5)	334 (80.3)
Male	3,678 (36.7)	2,651 (42.4)	259 (28.7)	459 (29.0)	227 (26.5)	82 (19.7)
**Race, *n* (%)**						
White	8,885 (88.8)	5,700 (91.2)	761 (84.3)	1,355 (85.7)	747 (87.1)	322 (77.4)
Non-White	1,126 (11.2)	552 (8.8)	142 (15.7)	227 (14.3)	111 (12.9)	94 (22.6)
**Highest degree in education, *n* (%)**						
High school and below	5,630 (56.2)	3,173 (50.8)	587 (65.0)	1,012 (64.0)	536 (62.5)	322 (77.4)
Some college	2,230 (22.3)	1,481 (23.7)	171 (18.9)	318 (20.1)	194 (22.6)	66 (15.9)
College and above	2,151 (21.5)	1,598 (25.6)	145 (16.1)	252 (15.9)	128 (14.9)	28 (6.7)
**Marital status, *n* (%)**						
Married or partnered	7,554 (75.5)	4,982 (79.7)	602 (66.7)	1,122 (70.9)	623 (72.6)	225 (54.1)
Separated/divorced/widowed/never married	2,457 (24.5)	1,270 (20.3)	301 (33.3)	460 (29.1)	235 (27.4)	191 (45.9)

Number of missing values before imputation: None.

**TABLE 2:** Initial health behaviors and conditions for analytical sample at the beginning of exposure period.

Variables	Overall sample	Depressive symptoms trajectory group				
		Consistently low	Decreasing	Fluctuating	Increasing	Consistently high
Individuals, *n* (%)	10,011	6,252 (62.5)	903 (9.0)	1,582 (15.8)	858 (8.6)	416 (4.1)
**Health behaviors**						
BMI, kg/m^2^, mean (SD)	27.7 (5.0)	27.3 (4.7)	28.1 (5.3)	28.1 (5.2)	28.4 (5.3)	29.4 (6.0)
**Drinking status, *n* (%)**						
Never drinkers	3,337 (33.3)	1,816 (29.0)	339 (37.6)	611 (38.6)	351 (40.9)	220 (52.9)
Ever drinkers	6,673 (66.7)	4,436 (71.0)	563 (62.4)	971 (61.4)	507 (59.1)	196 (47.1)
**Smoking status, *n* (%)**						
Never smokers	4,455 (44.7)	2,834 (45.5)	390 (43.4)	701 (44.6)	362 (42.5)	168 (40.5)
Ever smokers	5,502 (55.3)	3,388 (54.5)	508 (56.6)	869 (55.4)	490 (57.5)	247 (59.5)
**Vigorous exercise, *n* (%)**						
More than once per week	2,632 (26.3)	1,893 (30.3)	197 (21.8)	335 (21.2)	165 (19.2)	42 (10.1)
Once per week or 1–3 times per month	1,984 (19.8)	1,339 (21.4)	162 (17.9)	274 (17.3)	151 (17.6)	58 (13.9)
Never	5,395 (53.9)	3,020 (48.3)	544 (60.2)	973 (61.5)	542 (63.2)	316 (76.0)
**Health conditions**						
Heart condition (yes/no), *n* (%)	923 (9.2)	522 (8.3)	94 (10.4)	170 (10.7)	85 (9.9)	52 (12.5)
Hypertension (yes/no), *n* (%)	3,199 (32.0)	1,849 (29.6)	327 (36.2)	523 (33.1)	325 (37.9)	175 (42.1)
Diabetes (yes/no), *n* (%)	633 (6.3)	311 (5.0)	72 (8.0)	123 (7.8)	85 (9.9)	42 (10.1)
Cancer (yes/no), *n* (%)	585 (5.8)	374 (6.0)	43 (4.8)	90 (5.7)	51 (5.9)	27 (6.5)

BMI stands for body mass index. The percentage of missing values before imputation is as follows: BMI 3.97%, smoking 0.54%, drinking 0.01%, and cancer 0.01%. There are no missing values for the other variables.

**FIGURE 3:**
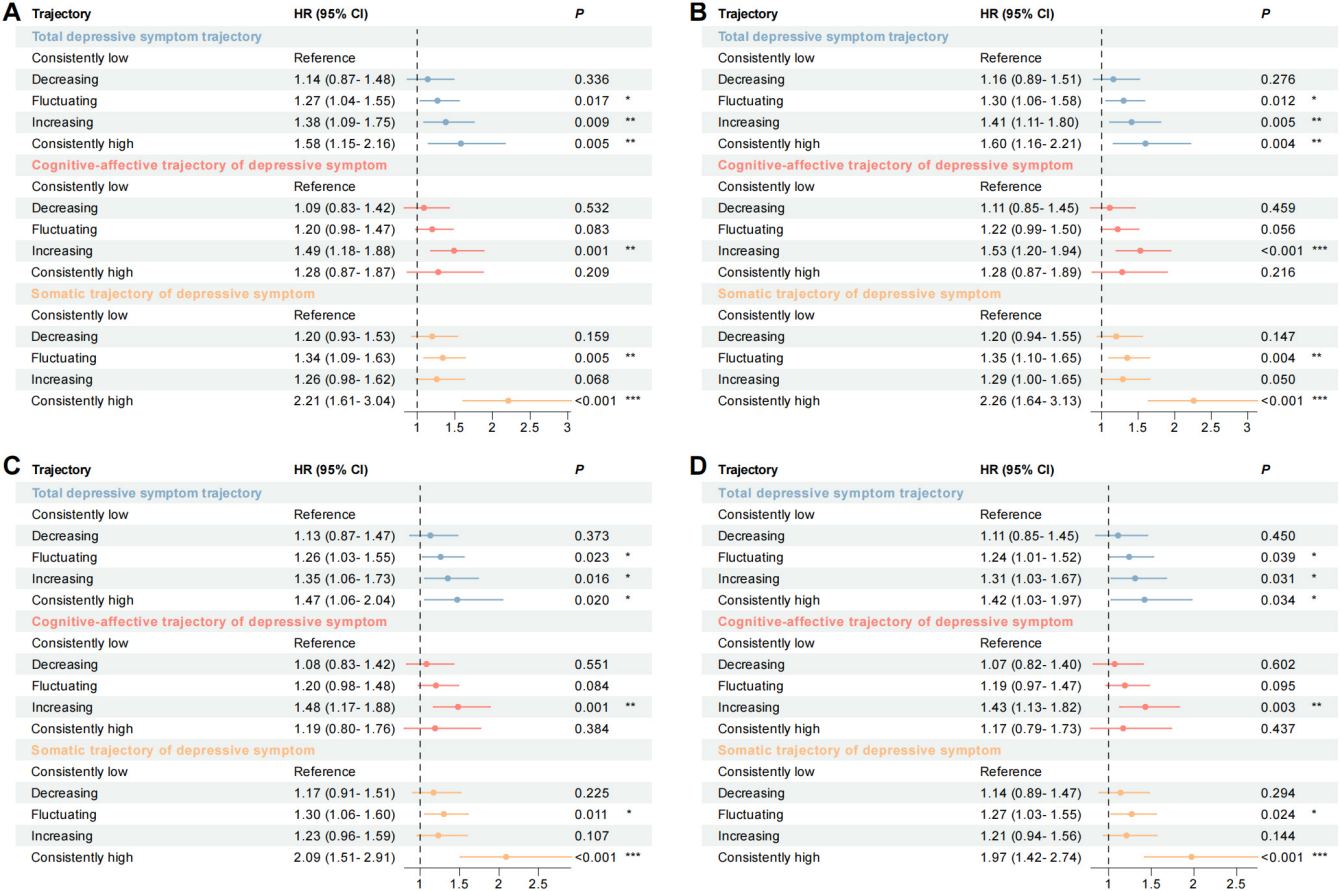
Forest plot depicting the cox proportional hazard ratios found in Table 3. This forest plot visually displays the hazard ratios (HRs) and 95% confidence intervals (CIs) for each depressive symptom trajectory across various models in Table 3. Every horizontal line is linked to a particular trajectory group. The square symbolizes the point estimate (HR), and the horizontal line shows the 95% CI. The vertical dashed line signifies the null value (HR = 1), which means no effect. Significant causal associations are designated with asterisk (*P-value < 0.05; **P-value < 0.01; ***P-value < 0.001). **(A)** Model 1 adjusts for age only. **(B)** Model 2 additionally adjusts for sociodemographics (race, education, marital status, and sex). **(C)** Model 3 additionally adjusts for health behaviors (vigorous exercise, alcohol consumption, BMI, and smoking status). **(D)** Model 4 additionally adjusts for health conditions (hypertension, heart conditions, diabetes, and cancer).

Table 3 and Figure 3 illustrate longitudinal relationships between trajectories of total, cognitive-affective, and somatic depressive symptoms and incident stroke risk. In the fully adjusted Model 4 (demographics, health behaviors, health conditions), individuals with a consistently high total depressive symptom trajectory showed significantly elevated stroke risk versus consistently low (HR = 1.42, 95% CI: 1.03–1.97). Similarly, relative to reference, individuals with increasing (HR = 1.31, 95% CI: 1.03–1.67) and fluctuating (HR = 1.24, 95% CI: 1.01–1.52) trajectories also exhibited heightened risk. Conversely, the decreasing trajectory showed no statistically significant difference in risk (HR = 1.11, 95% CI: 0.85–1.45).

**TABLE 3:** Cox proportional hazard ratios examining the relationship between depressive symptom trajectories and incident stroke over a decade for the whole sample.

		Model 1^†^		Model 2^‡^		Model 3^§^		Model 4^¶^	
Depressive symptoms trajectory group	No. of cases (%)	HR (95% CI)	*P*-value	HR (95% CI)	*P*-value	HR (95% CI)	*P*-value	HR (95% CI)	*P*-value
**Total depressive symptom trajectory**									
Consistently low	6,252 (62.5)	Reference		Reference		Reference		Reference	
Decreasing	903 (9.0)	1.14 (0.87–1.48)	0.336	1.16 (0.89–1.51)	0.276	1.13 (0.87–1.47)	0.373	1.11 (0.85–1.45)	0.450
Fluctuating	1,582 (15.8)	1.27 (1.04–1.55)	0.017*	1.30 (1.06–1.58)	0.012*	1.26 (1.03–1.55)	0.023*	1.24 (1.01–1.52)	0.039*
Increasing	858 (8.6)	1.38 (1.09–1.75)	0.009**	1.41 (1.11–1.80)	0.005**	1.35 (1.06–1.73)	0.016*	1.31 (1.03–1.67)	0.031*
Consistently high	416 (4.1)	1.58 (1.15–2.16)	0.005**	1.60 (1.16–2.21)	0.004**	1.47 (1.06–2.04)	0.020*	1.42 (1.03–1.97)	0.034*
**Cognitive-affective trajectory of depressive symptom**									
Consistently low	6,493 (64.9)	Reference		Reference		Reference		Reference	
Decreasing	894 (8.9)	1.09 (0.83–1.42)	0.532	1.11 (0.85–1.45)	0.459	1.08 (0.83–1.42)	0.551	1.07 (0.82–1.40)	0.602
Fluctuating	1,491 (14.9)	1.20 (0.98–1.47)	0.083	1.22 (0.99–1.50)	0.056	1.20 (0.98–1.48)	0.084	1.19 (0.97–1.47)	0.095
Increasing	833 (8.3)	1.49 (1.18–1.88)	0.001**	1.53 (1.20–1.94)	< 0.001***	1.48 (1.17–1.88)	0.001**	1.43 (1.13–1.82)	0.003**
Consistently high	300 (3.0)	1.28 (0.87–1.87)	0.209	1.28 (0.87–1.89)	0.216	1.19 (0.80–1.76)	0.384	1.17 (0.79–1.73)	0.437
**Somatic trajectory of depressive symptom**									
Consistently low	6,361 (63.6)	Reference		Reference		Reference		Reference	
Decreasing	994 (9.9)	1.20 (0.93–1.53)	0.159	1.20 (0.94–1.55)	0.147	1.17 (0.91–1.51)	0.225	1.14 (0.89–1.47)	0.294
Fluctuating	1,465 (14.6)	1.34 (1.09–1.63)	0.005**	1.35 (1.10–1.65)	0.004**	1.30 (1.06–1.60)	0.011*	1.27 (1.03–1.55)	0.024*
Increasing	872 (8.7)	1.26 (0.98–1.62)	0.068	1.29 (1.00–1.65)	0.050	1.23 (0.96–1.59)	0.107	1.21 (0.94–1.56)	0.144
Consistently high	319 (3.2)	2.21 (1.61–3.04)	< 0.001***	2.26 (1.64–3.13)	< 0.001***	2.09 (1.51–2.91)	< 0.001***	1.97 (1.42–2.74)	< 0.001***

† Model 1 adjusts for age only.

‡ Model 2 additionally adjusts for sociodemographics (race, education, marital status, and sex).

^§^Model 3 additionally adjusts for health behaviors (vigorous exercise, alcohol consumption, BMI, and smoking status).

^¶^Model 4 additionally adjusts for health conditions (hypertension, heart conditions, diabetes, and cancer).

Significant causal associations are designated with asterisk (*P-value < 0.05; **P-value < 0.01; ***P-value < 0.001).

Stratifying total depressive symptoms into subtypes revealed distinct associations. In Model 4, individuals with an increasing cognitive-affective trajectory demonstrated significantly elevated stroke risk versus consistently low cognitive-affective (HR = 1.43, 95% CI: 1.13–1.82). Conversely, risks for decreasing (HR = 1.07, 95% CI: 0.82–1.40), fluctuating (HR = 1.19, 95% CI: 0.97–1.47), and consistently high (HR = 1.17, 95% CI: 0.79–1.73) cognitive-affective trajectories were non-significant relative to reference.

Similarly, using consistently low somatic trajectory as reference in Model 4, individuals with consistently high somatic trajectory demonstrated significantly elevated stroke risk (HR = 1.97, 95% CI: 1.42–2.74). Those with a fluctuating somatic trajectory also exhibited increased risk (HR = 1.27, 95% CI: 1.03–1.55). Conversely, increasing (HR = 1.21, 95% CI: 0.94–1.56) or decreasing (HR = 1.14, 95% CI: 0.89–1.47) somatic trajectories showed no statistically significant difference in risk.

Sensitivity analyses confirmed the consistency and robustness of primary findings. Six distinct methods were employed (detailed in Supplementary Tables 2–9). Analyses consistently showed that fluctuating, increasing, and consistently high total depressive symptom trajectories were associated with elevated stroke risk versus consistently low, while the decreasing trajectory showed no significant correlation. Additionally, increasing cognitive-affective symptoms, alongside fluctuating and consistently high somatic trajectories, were linked to heightened stroke risk, with consistently high somatic presenting the greatest risk.

## Discussion

This research examined the association between long-term depressive symptom patterns and incident stroke likelihood in US and UK middle-aged and older adults. The assessed trajectories were classified as: consistently low, decreasing, fluctuating, increasing, and consistently high. Results indicate that, irrespective of whether symptoms were categorized as total, cognitive-affective, or somatic, no significant association existed between stroke risk and the decreasing trajectory. Conversely, fluctuating, increasing, and consistently high total depressive symptom trajectories, along with the increasing cognitive-affective trajectory and fluctuating and consistently high somatic trajectories, were identified as significant stroke risk factors. Among these, the consistently high somatic trajectory demonstrated the strongest association. Critically, these findings persisted after adjusting for potential confounding variables affecting the trajectory-stroke relationship. Sensitivity analyses, incorporating modifications to models, trajectories, covariates, and extended follow-up, further substantiated result robustness. To our knowledge, this is the first study exploring the relationship between cognitive-affective and somatic depressive symptom trajectories and stroke risk.

Depressive symptoms exhibit dynamism and can persist long-term; consequently, longitudinal assessment of symptom progression and variation enables a more thorough evaluation of their impact on incident stroke. Our findings suggest that persistent depressive symptoms—whether progressively worsening, fluctuating, or chronically elevated—are linked to increased stroke risk. This association likely stems from cumulative cardiovascular damage inflicted by prolonged exposure to depressive states. Notably, if patients experience an extended remission period, their risk of stroke may not be elevated despite having previously undergone severe depressive states. Potential biological mechanisms linking recurrently elevated depressive symptoms to stroke include neuroendocrine dysregulation, platelet dysfunction, and immune-inflammatory responses (Pan et al., 2011). Specifically, this trajectory of depressive symptoms may activate the hypothalamic-pituitary-adrenal (HPA) axis, leading to sustained secretion of cortisol (Iob et al., 2020). This hormonal imbalance is hypothesized to drive metabolic dysregulation and long-term physiological changes, such as atherosclerosis and white matter hyperintensities (Gilsanz et al., 2017). Ultimately, these cumulative pathological processes contribute to a significantly increased risk of incident stroke. Meta-analyses indicate that depressive symptom alleviation post-treatment correlates with reduced inflammatory markers, notably interleukin-6 (IL-6) and C-reactive protein (CRP) (Hiles et al., 2012; Strawbridge et al., 2015). This implies inflammation may be a critical mediator between depression and stroke, suggesting that modulating inflammation via depression treatment could aid stroke prevention. Some studies suggest antidepressant therapies may reduce stroke risk by attenuating inflammation (Zuzarte et al., 2018). Furthermore, depressive symptoms can be categorized into cognitive-affective and somatic subtypes.

Cognitive-affective symptoms (e.g., mood disturbances, anxiety, emotional stress) may elevate stroke risk through diverse mechanisms. Psychological stress from environmental factors like noise significantly impacts cardiovascular health (Min et al., 2024; Patten, 2012; Pendlebury, 2012). Chronic stress induces hormonal imbalances causing oxidative stress, damaging vasculature and increasing stroke risk (Brooks et al., 2018; Hahad et al., 2019; Lian et al., 2025). Additionally, cognitive-affective symptoms may foster poor health behaviors (reduced activity, unhealthy diet, poor treatment adherence). Depression can also engender loneliness and social isolation, creating a vicious cycle of poor health behaviors and heightened stroke risk (Valtorta et al., 2016). Our study found that individuals with an increasing cognitive-affective symptom trajectory had a higher risk of stroke, indicating that compared to decreasing or fluctuating trajectories, sustained and escalating psychological stress is more likely to induce pathological cerebral vascular changes and promote unhealthy lifestyles. This progressively worsening psychological pressure may create an environment where recovery seems unattainable. The unremitting upward trajectory of cognitive-affective symptoms exerts a stronger negative impact on cerebrovascular health. Therefore, we should be especially vigilant about the increased risk of stroke associated with rising cognitive-affective depressive symptoms.

In contrast, somatic depressive symptoms (e.g., energy depletion, sleep disturbances, psychomotor retardation) appear to possess stronger predictive power for stroke risk. These symptoms may increase risk through multiple pathways. Somatic symptoms can precipitate unhealthy behaviors [smoking (Ambrose and Barua, 2004; Ding et al., 2019; Middlekauff et al., 2014), excessive alcohol consumption (Biddinger et al., 2022; Csengeri et al., 2021; Whitman et al., 2017)], detrimental to cardiovascular health. Prolonged somatic symptoms may disrupt autonomic nervous system function, particularly by activating the sympathetic nervous system (Penninx, 2017), elevating heart rate and blood pressure, ultimately raising stroke risk. Additionally, fatigue—common in somatic depression—has been linked to increased stroke risk (Barlas et al., 2020). Sleep disturbances associated with somatic symptoms can elevate inflammatory markers (CRP, IL-6) (Kappelmann et al., 2021; Mayer-Suess et al., 2024), contributing to vascular damage and atherosclerosis, thereby increasing stroke risk (Kong et al., 2022; Soehnlein and Libby, 2021). This aligns with prior research showing depression treatment reduces inflammation (Strawbridge et al., 2015). Our study found fluctuating and consistently high somatic trajectories were strongly associated with stroke risk, with consistently high showing the most significant link. This suggests long-term somatic depressive symptoms, especially those that fluctuate or persist, are more likely to detrimentally impact cardiovascular health compared to shorter-term trajectories like increasing or decreasing. We believe that multiple factors jointly mediate the increased stroke risk associated with somatic depressive symptoms. While the impact of increasing cognitive-affective symptoms on stroke risk may be attributed to their escalating psychological burden, the effect of somatic symptoms appears to depend more on duration. The longer the persistence of somatic depressive symptoms, the greater the risk of stroke.

Additional mechanisms may underlie the observed dynamic alterations in depressive symptom trajectories and their stroke risk association. For example, the fluctuating trajectory group might include individuals diagnosed with bipolar disorder or other episodic mood disorders, possessing genetic profiles distinct from unipolar depression (Wassertheil-Smoller et al., 2018). The relationship between these conditions and stroke risk may be attributed to factors like side effects of mood stabilizers (vs. the disorder itself), medication-induced weight gain/metabolic abnormalities, and healthcare access challenges (Chen et al., 2019; Prieto et al., 2014). Moreover, individuals classified into distinct trajectory groups often display differing baseline characteristics, indicative of subpopulations (Byers et al., 2012). Progressive depressive symptoms might signal early-stage specific diseases (e.g., dementia) or represent a delayed depressive response to other physical conditions (hypertension, hypercholesterolemia, diabetes), all significantly associated with subsequent stroke risk (Fiske et al., 2009; Mirza et al., 2014). Consequently, we posit that diverse depressive symptom trajectories reflect not only distinct clinical profiles and potential pathological pathways but also varied origins of stroke risk. Tailored prevention and intervention strategies for these subgroups are imperative.

Prior to this investigation, limited research explored the relationship between depressive symptom trajectories and incident stroke (Li et al., 2022; Loyal et al., 2025; Soh et al., 2022; Wu et al., 2022), with no studies differentiating subtypes. Moreover, existing studies typically defined trajectories based on only two assessment cycles. This study’s strength lies in integrating representative prospective cohorts (HRS, ELSA), utilizing repeated symptom assessments and long-term follow-up, while incorporating a comprehensive covariate set to control confounding. Extensive sensitivity analyses bolstered the findings’ robustness and generalizability. Additionally, distinguishing between depressive symptom subtypes and delineating long-term dynamic trajectories for both total and subtype-specific symptoms enabled identification of differential stroke risks associated with specific subtypes and trajectories, offering nuanced explanations for the depression-stroke association. Furthermore, participant selection was not contingent on mental health issues, enhancing sample representativeness relative to the general population. Nonetheless, limitations exist. First, while the somatic symptom classification derived from the CES-D scale is contextually appropriate, it may not perfectly align with definitions in other scholarly works or measurement tools (Shafer, 2006). Second, excluding individuals lacking CES-D scores or experiencing stroke pre-exposure limits generalizability to these populations. Third, focusing on individuals ≥ 45 years may limit applicability to younger cohorts. Fourth, reliance on self-reported questionnaires introduces potential recall bias, possibly compromising response accuracy. Fifth, medication adherence could influence the trajectory-stroke risk relationship; however, adherence data (accessible primarily via pharmacy records) were unavailable in this study. Therefore, results should be interpreted cautiously, acknowledging these unaccounted confounding factors.

## Conclusion

This study’s findings suggest that recurrently elevated total depressive symptoms, characterized by increasing, fluctuating, or persistently high trajectories, are associated with heightened incident stroke risk. However, stroke risk appears modifiable with symptom changes, as individuals exhibiting decreasing symptoms show risk comparable to those with consistently low symptoms. Worsening, unstable, or chronically high total depressive symptoms may signal underlying cerebrovascular issues. Examining subtypes revealed that an ascending trajectory of cognitive-affective symptoms, alongside unstable and persistently elevated trajectories of somatic symptoms, are associated with increased stroke risk. These insights provide crucial clinical guidance for stroke prevention. Future research should delve into the overarching and trajectory-specific mechanisms underpinning these associations to enhance identification and protection of high-risk populations.

## Data Availability

The datasets utilized in this research are accessible to the public via the Health and Retirement Study (HRS) database (https://hrs.isr.umich.edu) and the English Longitudinal Study of Ageing (ELSA) database (www.elsa-project.ac.uk). The HRS is supported by the National Institute on Aging under grant number NIA U01AG009740 and is administered by the University of Michigan. Similarly, the ELSA is funded by the National Institute on Aging of the National Institutes of Health, with award number R01AG017644, as well as by various UK Government Departments, including the Department of Health and Social Care (DHSC), the Department for Transport (DfT), and the Department for Work and Pensions (DWP), and is coordinated through the National Institute for Health and Care Research Health and Social Care (NIHRHR) Policy Research Programme.
