# Supplementary material for: The association of eight-year trajectories in total, cognitive-affective, and somatic depressive symptoms with incident stroke: a 10-year follow-up study using HRS and ELSA cohorts
Source: Front Aging Neurosci. 2026 Jan 9;17:1733007. doi: 10.3389/fnagi.2025.1733007 (PMC12827575; doi:10.3389/fnagi.2025.1733007)
Supplement: Supplementary file 1 [file Table_1.docx]

**Supplementary Table 1.** Trajectory Definitions of Depressive Symptoms

|  | First wave | Second wave | Third wave | Fourth wave |
| --- | --- | --- | --- | --- |
| Consistently low | **-** | **-** | **-** | **-** |
| Consistently high | **+** | **+** | **+** | **+** |
| Decreasing | **+** | **-** | **-** | **-** |
|  | **+** | **+** | **-** | **-** |
| Increasing | **-** | **+** | **+** | **+** |
|  | **-** | **-** | **+** | **+** |
| Fluctuating | All other cases where the above conditions are not met | | | |

**Supplementary Table 2.** Hazard Ratios from Discrete-Time Models for the Association of Depressive Symptom Trajectories with Incident Stroke

|  |  | **Model 3 ^§^** |  | **Model 4 ^¶^** |  |
| --- | --- | --- | --- | --- | --- |
|  | **No. of cases (%)** | **HR (95% CI)** | ***P*-value** | **HR (95% CI)** | ***P*-value** |
| *Total depressive symptom trajectory* | | | | | |
| Consistently low | 6252 (62.5) | Reference |  | Reference |  |
| Decreasing | 903 (9.0) | 1.18 (0.90- 1.53) | 0.226 | 1.11 (0.84- 1.44) | 0.444 |
| Fluctuating | 1582 (15.8) | 1.33 (1.08- 1.62) | 0.006**^**^** | 1.26 (1.03- 1.54) | 0.024**^*^** |
| Increasing | 858 (8.6) | 1.44 (1.12- 1.83) | 0.004**^**^** | 1.33 (1.03- 1.69) | 0.024**^*^** |
| Consistently high | 416 (4.1) | 1.64 (1.17- 2.24) | 0.003**^**^** | 1.45 (1.03- 1.99) | 0.027**^*^** |
| *Cognitive-affective trajectory of depressive symptom* | | | | | |
| Consistently low | 6493 (64.9) | Reference |  | Reference |  |
| Decreasing | 894 (8.9) | 1.13 (0.85- 1.46) | 0.389 | 1.08 (0.82- 1.41) | 0.573 |
| Fluctuating | 1491 (14.9) | 1.24 (1.01- 1.53) | 0.039**^*^** | 1.21 (0.98- 1.49) | 0.068 |
| Increasing | 833 (8.3) | 1.56 (1.22- 1.97) | <0.001**^***^** | 1.45 (1.13- 1.84) | 0.002**^**^** |
| Consistently high | 300 (3.0) | 1.35 (0.89- 1.96) | 0.137 | 1.22 (0.81- 1.78) | 0.318 |
| *Somatic trajectory of depressive symptom* | | | | | |
| Consistently low | 6361 (63.6) | Reference |  | Reference |  |
| Decreasing | 994 (9.9) | 1.22 (0.94- 1.56) | 0.122 | 1.14 (0.88- 1.47) | 0.298 |
| Fluctuating | 1465 (14.6) | 1.37 (1.11- 1.67) | 0.003**^**^** | 1.27 (1.03- 1.56) | 0.022**^*^** |
| Increasing | 872 (8.7) | 1.29 (1.00- 1.65) | 0.048**^*^** | 1.20 (0.93- 1.54) | 0.156 |
| Consistently high | 319 (3.2) | 2.33 (1.66- 3.20) | <0.001**^***^** | 2.01 (1.42- 2.77) | <0.001**^***^** |

**^§^** Model 3 adjusts for sociodemographics (age, race, education, marital status, and sex) and health behaviors (vigorous exercise, alcohol consumption, BMI, and smoking status). **^¶^** Model 4 additionally adjusts for health conditions (hypertension, heart conditions, diabetes, and cancer). Significant causal associations are designated with asterisk (**^*^***P*-value<0.05; **^**^***P*-value<0.01; **^***^***P*-value<0.001).

**Supplementary Table 3.** Odds Ratios from Logistic Regression Models for the Association of Depressive Symptom Trajectories with Incident Stroke

|  |  | **Model 3 ^§^** |  | **Model 4 ^¶^** |  |
| --- | --- | --- | --- | --- | --- |
|  | **No. of cases (%)** | **OR (95% CI)** | ***P*-value** | **OR (95% CI)** | ***P*-value** |
| *Total depressive symptom trajectory* | | | | | |
| Consistently low | 6252 (62.5) | Reference |  | Reference |  |
| Decreasing | 903 (9.0) | 1.20 (0.90- 1.57) | 0.197 | 1.12 (0.84- 1.48) | 0.414 |
| Fluctuating | 1582 (15.8) | 1.36 (1.10- 1.68) | 0.004**^**^** | 1.29 (1.04- 1.59) | 0.019**^*^** |
| Increasing | 858 (8.6) | 1.50 (1.15- 1.93) | 0.002**^**^** | 1.38 (1.06- 1.78) | 0.016**^*^** |
| Consistently high | 416 (4.1) | 1.74 (1.22- 2.43) | 0.002**^**^** | 1.52 (1.06- 2.13) | 0.019**^*^** |
| *Cognitive-affective trajectory of depressive symptom* | | | | | |
| Consistently low | 6493 (64.9) | Reference |  | Reference |  |
| Decreasing | 894 (8.9) | 1.15 (0.86- 1.51) | 0.339 | 1.10 (0.82- 1.44) | 0.528 |
| Fluctuating | 1491 (14.9) | 1.27 (1.02- 1.57) | 0.031**^*^** | 1.24 (0.99- 1.53) | 0.058 |
| Increasing | 833 (8.3) | 1.61 (1.24- 2.07) | <0.001**^***^** | 1.50 (1.16- 1.93) | 0.002**^**^** |
| Consistently high | 300 (3.0) | 1.45 (0.94- 2.15) | 0.081 | 1.30 (0.84- 1.94) | 0.217 |
| *Somatic trajectory of depressive symptom* | | | | | |
| Consistently low | 6361 (63.6) | Reference |  | Reference |  |
| Decreasing | 994 (9.9) | 1.25 (0.95- 1.62) | 0.098 | 1.16 (0.88- 1.51) | 0.271 |
| Fluctuating | 1465 (14.6) | 1.41 (1.14- 1.74) | 0.002**^**^** | 1.31 (1.05- 1.62) | 0.016**^*^** |
| Increasing | 872 (8.7) | 1.32 (1.01- 1.71) | 0.040**^*^** | 1.23 (0.93- 1.59) | 0.138 |
| Consistently high | 319 (3.2) | 2.46 (1.71- 3.46) | <0.001**^***^** | 2.09 (1.44- 2.96) | <0.001**^***^** |

**^§^** Model 3 adjusts for sociodemographics (age, race, education, marital status, and sex) and health behaviors (vigorous exercise, alcohol consumption, BMI, and smoking status). **^¶^** Model 4 additionally adjusts for health conditions (hypertension, heart conditions, diabetes, and cancer). Significant causal associations are designated with asterisk (**^*^***P*-value<0.05; **^**^***P*-value<0.01; **^***^***P*-value<0.001).

**Supplementary Table 4.** Cox Proportional Hazard Ratios for the Association of Depressive Symptom Trajectories with Incident Stroke Over a 10-year Follow-up Period for the Overall Sample, Adjusted for Decreasing and Increasing Trajectory Groups

|  |  | **Model 3 ^§^** |  | **Model 4 ^¶^** |  |
| --- | --- | --- | --- | --- | --- |
|  | **No. of cases (%)** | **HR (95% CI)** | ***P*-value** | **HR (95% CI)** | ***P*-value** |
| *Total depressive symptom trajectory* | | | | | |
| Consistently low | 6252 (62.5) | Reference |  | Reference |  |
| Decreasing | 750 (7.5) | 1.10 (0.83- 1.48) | 0.504 | 1.08 (0.81- 1.45) | 0.587 |
| Fluctuating | 2133 (21.3) | 1.25 (1.04- 1.50) | 0.016**^*^** | 1.22 (1.02- 1.47) | 0.031**^*^** |
| Increasing | 460 (4.6) | 1.49 (1.10- 2.02) | 0.011**^*^** | 1.44 (1.06- 1.96) | 0.019**^*^** |
| Consistently high | 416 (4.1) | 1.47 (1.06- 2.04) | 0.020**^*^** | 1.42 (1.03- 1.97) | 0.034**^*^** |
| *Cognitive-affective trajectory of depressive symptom* | | | | | |
| Consistently low | 6493 (64.9) | Reference |  | Reference |  |
| Decreasing | 770 (7.7) | 1.12 (0.85- 1.49) | 0.424 | 1.11 (0.83- 1.47) | 0.475 |
| Fluctuating | 2034 (20.3) | 1.20 (1.00- 1.45) | 0.051 | 1.19 (0.99- 1.43) | 0.067 |
| Increasing | 414 (4.1) | 1.65 (1.21- 2.24) | 0.001**^**^** | 1.60 (1.18- 2.18) | 0.003**^**^** |
| Consistently high | 300 (3.0) | 1.19 (0.81- 1.77) | 0.377 | 1.17 (0.79- 1.73) | 0.430 |
| *Somatic trajectory of depressive symptom* | | | | | |
| Consistently low | 6361 (63.5) | Reference |  | Reference |  |
| Decreasing | 843 (8.4) | 1.26 (0.97- 1.64) | 0.083 | 1.24 (0.95- 1.61) | 0.111 |
| Fluctuating | 2020 (20.2) | 1.25 (1.04- 1.50) | 0.019**^*^** | 1.21 (1.01- 1.46) | 0.043**^*^** |
| Increasing | 468 (4.7) | 1.21 (0.86- 1.68) | 0.270 | 1.20 (0.86- 1.67) | 0.294 |
| Consistently high | 319 (3.2) | 2.09 (1.51- 2.91) | <0.001**^***^** | 1.97 (1.42- 2.74) | <0.001**^***^** |

**^§^** Model 3 adjusts for sociodemographics (age, race, education, marital status, and sex) and health behaviors (vigorous exercise, alcohol consumption, BMI, and smoking status). **^¶^** Model 4 additionally adjusts for health conditions (hypertension, heart conditions, diabetes, and cancer). Significant causal associations are designated with asterisk (**^*^***P*-value<0.05; **^**^***P*-value<0.01; **^***^***P*-value<0.001).

**Supplementary Table 5.** Cox Proportional Hazard Ratios for the Association of Depressive Symptom Trajectories with Incident Stroke Over a 10-year Follow-up Period for the Overall Sample, Adjusted for Consistently High Trajectory Group

|  |  | **Model 1 ^†^** |  | **Model 2 ^‡^** |  | **Model 3 ^§^** |  | **Model 4 ^¶^** |  |
| --- | --- | --- | --- | --- | --- | --- | --- | --- | --- |
|  | **No. of cases (%)** | **HR (95% CI)** | ***P*-value** | **HR (95% CI)** | ***P*-value** | **HR (95% CI)** | ***P*-value** | **HR (95% CI)** | ***P*-value** |
| *Total depressive symptom trajectory* | | | | | | | | | |
| Consistently low | 6252 (62.5) | Reference |  | Reference |  | Reference |  | Reference |  |
| Decreasing | 969 (9.7) | 1.11 (0.86- 1.44) | 0.417 | 1.13 (0.87- 1.47) | 0.344 | 1.10 (0.85- 1.42) | 0.486 | 1.08 (0.83- 1.40) | 0.581 |
| Fluctuating | 1716 (17.1) | 1.32 (1.09- 1.59) | 0.004**^**^** | 1.34 (1.10- 1.63) | 0.003**^**^** | 1.30 (1.07- 1.58) | 0.007**^**^** | 1.28 (1.05- 1.55) | 0.014**^*^** |
| Increasing | 858 (8.6) | 1.38 (1.09- 1.75) | 0.009**^**^** | 1.41 (1.11- 1.80) | 0.005**^**^** | 1.35 (1.06- 1.72) | 0.016**^*^** | 1.31 (1.02- 1.67) | 0.032**^*^** |
| Consistently high | 216 (2.1) | 1.67 (1.10- 2.55) | 0.016**^*^** | 1.69 (1.10- 2.60) | 0.016**^*^** | 1.54 (1.00- 2.38) | 0.048**^*^** | 1.47 (0.95- 2.26) | 0.081 |

**^†^**Model 1 adjusts for age only. **^‡^**Model 2 additionally adjusts for sociodemographics (race, education, marital status, and sex). **^§^** Model 3 additionally adjusts for health behaviors (vigorous exercise, alcohol consumption, BMI, and smoking status). **^¶^**Model 4 additionally adjusts for health conditions (hypertension, heart conditions, diabetes, and cancer). Significant causal associations are designated with asterisk (**^*^***P*-value<0.05; **^**^***P*-value<0.01; **^***^***P*-value<0.001).

Note. Since we defined the Consistently high depressive symptom trajectory as a score of 4 or higher, which is only applicable for adjusting the overall depressive symptom trajectories, we conducted analyses only on the four models related to this part.

**Supplementary Table 6.** After adding income as a covariate, Cox proportional hazard ratios for the association of depressive symptom trajectories with incident stroke over a 10-year follow-up period for the overall sample

|  |  | **Model 1 ^†^** |  | **Model 2 ^‡^** |  | **Model 3 ^§^** |  | **Model 4 ^¶^** |  |
| --- | --- | --- | --- | --- | --- | --- | --- | --- | --- |
|  | **No. of cases (%)** | **HR (95% CI)** | ***P*-value** | **HR (95% CI)** | ***P*-value** | **HR (95% CI)** | ***P*-value** | **HR (95% CI)** | ***P*-value** |
| *Total depressive symptom trajectory* | | | | | | | | | |
| Consistently low | 6252 (62.5) | Reference |  | Reference |  | Reference |  | Reference |  |
| Decreasing | 903 (9.0) | 1.14 (0.87- 1.48) | 0.336 | 1.16 (0.89- 1.51) | 0.270 | 1.13 (0.87- 1.47) | 0.365 | 1.11 (0.85- 1.45) | 0.440 |
| Fluctuating | 1582 (15.8) | 1.27 (1.04- 1.55) | 0.017**^*^** | 1.30 (1.06- 1.59) | 0.011**^*^** | 1.27 (1.03- 1.55) | 0.022**^*^** | 1.24 (1.01- 1.52) | 0.037**^*^** |
| Increasing | 858 (8.6) | 1.38 (1.09- 1.75) | 0.009**^**^** | 1.41 (1.11- 1.80) | 0.005**^**^** | 1.35 (1.06- 1.73) | 0.015**^*^** | 1.31 (1.03- 1.68) | 0.030**^*^** |
| Consistently high | 416 (4.1) | 1.58 (1.15- 2.16) | 0.005**^**^** | 1.61 (1.16- 2.22) | 0.004**^**^** | 1.48 (1.07- 2.05) | 0.019**^*^** | 1.43 (1.03- 1.98) | 0.032**^*^** |
| *Cognitive-affective trajectory of depressive symptom* | | | | | | | | | |
| Consistently low | 6493 (64.9) | Reference |  | Reference |  | Reference |  | Reference |  |
| Decreasing | 894 (8.9) | 1.09 (0.83- 1.42) | 0.532 | 1.11 (0.85- 1.45) | 0.459 | 1.08 (0.83- 1.42) | 0.551 | 1.07 (0.82- 1.40) | 0.600 |
| Fluctuating | 1491 (14.9) | 1.20 (0.98- 1.47) | 0.083 | 1.22 (0.99- 1.50) | 0.056 | 1.20 (0.98- 1.48) | 0.084 | 1.19 (0.97- 1.47) | 0.094 |
| Increasing | 833 (8.3) | 1.49 (1.18- 1.88) | 0.001**^**^** | 1.53 (1.21- 1.94) | <0.001**^***^** | 1.48 (1.17- 1.88) | 0.001**^**^** | 1.44 (1.13- 1.82) | 0.003**^**^** |
| Consistently high | 300 (3.0) | 1.28 (0.87- 1.87) | 0.209 | 1.28 (0.87- 1.90) | 0.209 | 1.19 (0.81- 1.77) | 0.374 | 1.17 (0.79- 1.74) | 0.425 |
| *Somatic trajectory of depressive symptom* | | | | | | | | | |
| Consistently low | 6361 (63.6) | Reference |  | Reference |  | Reference |  | Reference |  |
| Decreasing | 994 (9.9) | 1.20 (0.93- 1.53) | 0.159 | 1.21 (0.94- 1.56) | 0.140 | 1.17 (0.91- 1.51) | 0.216 | 1.15 (0.89- 1.48) | 0.281 |
| Fluctuating | 1465 (14.6) | 1.34 (1.09- 1.63) | 0.005**^**^** | 1.35 (1.10- 1.66) | 0.003**^**^** | 1.31 (1.06- 1.60) | 0.011**^*^** | 1.27 (1.03- 1.56) | 0.023**^*^** |
| Increasing | 872 (8.7) | 1.26 (0.98- 1.62) | 0.068 | 1.29 (1.00- 1.66) | 0.049**^*^** | 1.23 (0.96 - 1.59) | 0.104 | 1.21 (0.94- 1.56) | 0.140 |
| Consistently high | 319 (3.2) | 2.21 (1.61- 3.04) | <0.001**^***^** | 2.28 (1.65- 3.15) | <0.001**^***^** | 2.11 (1.52- 2.93) | <0.001**^***^** | 1.99 (1.43- 2.77) | <0.001**^***^** |

**^†^**Model 1 adjusts for age only. **^‡^**Model 2 additionally adjusts for sociodemographics (race, education, marital status, and sex). **^§^** Model 3 additionally adjusts for health behaviors (vigorous exercise, alcohol consumption, BMI, and smoking status). **^¶^**Model 4 additionally adjusts for health conditions (hypertension, heart conditions, diabetes, and cancer). Significant causal associations are designated with asterisk (**^*^***P*-value<0.05; **^**^***P*-value<0.01; **^***^***P*-value<0.001).

**Supplementary Table 7.** Cox Proportional Hazard Ratios for the Association of Depressive Symptom Trajectories with Incident Stroke Over a 12-year Follow-up Period in the HRS Database

|  |  | **Model 3 ^§^** |  | **Model 4 ^¶^** |  |
| --- | --- | --- | --- | --- | --- |
|  | **No. of cases (%)** | **HR (95% CI)** | ***P*-value** | **HR (95% CI)** | ***P*-value** |
| *Total depressive symptom trajectory* | | | | | |
| Consistently low | 3736 (63.1) | Reference |  | Reference |  |
| Decreasing | 450 (7.6) | 1.07 (0.81- 1.40) | 0.645 | 1.05 (0.80- 1.38) | 0.741 |
| Fluctuating | 1220 (20.6) | 1.27 (1.04- 1.56) | 0.021**^*^** | 1.24 (1.01- 1.52) | 0.042**^*^** |
| Increasing | 271 (4.6) | 1.32 (1.03- 1.71) | 0.031**^*^** | 1.27 (0.99- 1.65) | 0.063 |
| Consistently high | 242 (4.1) | 1.53 (1.09- 2.14) | 0.015**^*^** | 1.47 (1.04- 2.06) | 0.028**^*^** |
| *Cognitive-affective trajectory of depressive symptom* | | | | | |
| Consistently low | 3803 (64.3) | Reference |  | Reference |  |
| Decreasing | 539 (9.1) | 1.05 (0.78- 1.43) | 0.730 | 1.04 (0.77- 1.41) | 0.795 |
| Fluctuating | 897 (15.1) | 1.15 (0.90- 1.46) | 0.256 | 1.13 (0.89- 1.43) | 0.332 |
| Increasing | 498 (8.4) | 1.54 (1.18- 2.01) | 0.001**^**^** | 1.47 (1.13- 1.92) | 0.004**^**^** |
| Consistently high | 182 (3.1) | 1.19 (0.75- 1.89) | 0.454 | 1.16 (0.73- 1.84) | 0.524 |
| *Somatic trajectory of depressive symptom* | | | | | |
| Consistently low | 3827 (64.7) | Reference |  | Reference |  |
| Decreasing | 564 (9.5) | 1.04 (0.77- 1.41) | 0.786 | 1.02 (0.76- 1.38) | 0.881 |
| Fluctuating | 835 (14.1) | 1.35 (1.07- 1.70) | 0.011**^*^** | 1.31 (1.04- 1.66) | 0.021**^*^** |
| Increasing | 510 (8.6) | 1.20 (0.90- 1.61) | 0.208 | 1.19 (0.89- 1.58) | 0.248 |
| Consistently high | 183 (3.1) | 2.21 (1.52- 3.22) | <0.001**^***^** | 2.04 (1.40- 2.98) | <0.001**^***^** |

**^§^** Model 3 adjusts for sociodemographics (age, race, education, marital status, and sex) and health behaviors (vigorous exercise, alcohol consumption, BMI, and smoking status). **^¶^** Model 4 additionally adjusts for health conditions (hypertension, heart conditions, diabetes, and cancer). Significant causal associations are designated with asterisk (**^*^***P*-value<0.05; **^**^***P*-value<0.01; **^***^***P*-value<0.001).

**Supplementary Table 8.** Cox Proportional Hazard Ratios for the Association of Depressive Symptom Trajectories with Incident Stroke Over a 14-year Follow-up Period in the HRS Database

|  |  | **Model 3 ^§^** |  | **Model 4 ^¶^** |  |
| --- | --- | --- | --- | --- | --- |
|  | **No. of cases (%)** | **HR (95% CI)** | ***P*-value** | **HR (95% CI)** | ***P*-value** |
| *Total depressive symptom trajectory* | | | | | |
| Consistently low | 3042 (64.5) | Reference |  | Reference |  |
| Decreasing | 396 (8.4) | 1.20 (0.90- 1.59) | 0.207 | 1.17 (0.88- 1.56) | 0.273 |
| Fluctuating | 727 (15.4) | 1.25 (1.00- 1.55) | 0.051 | 1.22 (0.98- 1.52) | 0.079 |
| Increasing | 365 (7.7) | 1.24 (0.93- 1.66) | 0.139 | 1.20 (0.90- 1.60) | 0.221 |
| Consistently high | 189 (4.0) | 1.55 (1.09- 2.22) | 0.015**^*^** | 1.50 (1.05- 2.15) | 0.026**^*^** |
| *Cognitive-affective trajectory of depressive symptom* | | | | | |
| Consistently low | 3080 (65.3) | Reference |  | Reference |  |
| Decreasing | 408 (8.6) | 1.11 (0.81- 1.53) | 0.517 | 1.10 (0.80- 1.51) | 0.571 |
| Fluctuating | 709 (15.0) | 1.07 (0.82- 1.39) | 0.631 | 1.04 (0.80- 1.35) | 0.765 |
| Increasing | 381 (8.1) | 1.46 (1.08- 1.97) | 0.014**^*^** | 1.40 (1.04- 1.90) | 0.029**^*^** |
| Consistently high | 141 (3.0) | 0.94 (0.55- 1.60) | 0.827 | 0.92 (0.54- 1.56) | 0.753 |
| *Somatic trajectory of depressive symptom* | | | | | |
| Consistently low | 3122 (66.2) | Reference |  | Reference |  |
| Decreasing | 420 (8.9) | 0.98 (0.69- 1.39) | 0.921 | 0.98 (0.69- 1.38) | 0.888 |
| Fluctuating | 650 (13.8) | 1.48 (1.16- 1.90) | 0.002**^**^** | 1.46 (1.14- 1.87) | 0.003**^**^** |
| Increasing | 380 (8.1) | 1.38 (1.00- 1.88) | 0.047**^*^** | 1.35 (0.98- 1.85) | 0.062 |
| Consistently high | 147 (3.0) | 2.33 (1.55- 3.49) | <0.001**^***^** | 2.17 (1.44- 3.25) | <0.001**^***^** |

**^§^** Model 3 adjusts for sociodemographics (age, race, education, marital status, and sex) and health behaviors (vigorous exercise, alcohol consumption, BMI, and smoking status). **^¶^** Model 4 additionally adjusts for health conditions (hypertension, heart conditions, diabetes, and cancer). Significant causal associations are designated with asterisk (**^*^***P*-value<0.05; **^**^***P*-value<0.01; **^***^***P*-value<0.001).

**Supplementary Table 9.** Cox Proportional Hazard Ratios for the Association of Depressive Symptom Trajectories with Incident Stroke Over a 16-year Follow-up Period in the HRS Database

|  |  | **Model 3 ^§^** |  | **Model 4 ^¶^** |  |
| --- | --- | --- | --- | --- | --- |
|  | **No. of cases (%)** | **HR (95% CI)** | ***P*-value** | **HR (95% CI)** | ***P*-value** |
| *Total depressive symptom trajectory* | | | | | |
| Consistently low | 2490 (64.7) | Reference |  | Reference |  |
| Decreasing | 320 (8.3) | 1.15 (0.85- 1.55) | 0.379 | 1.12 (0.83- 1.52) | 0.469 |
| Fluctuating | 602 (15.6) | 1.24 (0.99- 1.56) | 0.065 | 1.22 (0.97- 1.54) | 0.084 |
| Increasing | 301 (7.8) | 1.27 (0.94- 1.71) | 0.117 | 1.24 (0.92- 1.68) | 0.156 |
| Consistently high | 137 (3.6) | 1.65 (1.13- 2.42) | 0.010**^*^** | 1.60 (1.09- 2.34) | 0.016**^*^** |
| *Cognitive-affective trajectory of depressive symptom* | | | | | |
| Consistently low | 2517 (65.4) | Reference |  | Reference |  |
| Decreasing | 335 (8.7) | 1.01 (0.71- 1.44) | 0.954 | 0.99 (0.69- 1.41) | 0.946 |
| Fluctuating | 582 (15.1) | 1.00 (0.76- 1.33) | 0.973 | 0.99 (0.75- 1.31) | 0.945 |
| Increasing | 316 (8.2) | 1.58 (1.16- 2.15) | 0.003**^**^** | 1.58 (1.16- 2.16) | 0.004**^**^** |
| Consistently high | 100 (2.6) | 1.12 (0.64- 1.99) | 0.687 | 1.06 (0.60- 1.88) | 0.833 |
| *Somatic trajectory of depressive symptom* | | | | | |
| Consistently low | 2590 (67.3) | Reference |  | Reference |  |
| Decreasing | 346 (9.0) | 0.98 (0.69- 1.41) | 0.931 | 0.97 (0.68- 1.39) | 0.871 |
| Fluctuating | 512 (13.3) | 1.27 (0.97- 1.68) | 0.085 | 1.26 (0.96- 1.66) | 0.103 |
| Increasing | 295 (7.6) | 1.24 (0.87- 1.76) | 0.229 | 1.24 (0.87- 1.75) | 0.237 |
| Consistently high | 107 (2.8) | 2.13 (1.36- 3.31) | <0.001**^***^** | 2.04 (1.31- 3.19) | 0.002**^**^** |

**^§^** Model 3 adjusts for sociodemographics (age, race, education, marital status, and sex) and health behaviors (vigorous exercise, alcohol consumption, BMI, and smoking status). **^¶^** Model 4 additionally adjusts for health conditions (hypertension, heart conditions, diabetes, and cancer). Significant causal associations are designated with asterisk (**^*^***P*-value<0.05; **^**^***P*-value<0.01; **^***^***P*-value<0.001).
